# Effects of Painting-Based Art Interventions on Mental Health Outcomes: A Meta-Analysis of Randomized Controlled Trials

**DOI:** 10.3390/bs16050830

**Published:** 2026-05-21

**Authors:** Xu Song, Jihoon Jang

**Affiliations:** Global Fine Arts Department, Kyonggi University, Suwon 16227, Republic of Korea; songxukgu@outlook.com

**Keywords:** art intervention, painting-based intervention, mental health, randomized controlled trials, meta-analysis, depression

## Abstract

Mental health issues, such as depression and anxiety, are rising globally, and while conventional therapies like medications and psychotherapy remain common, they face limitations, including side effects and accessibility. This highlights the need for effective non-pharmacological interventions. Painting-based art interventions are a promising non-pharmacological approach for improving scale-assessed mental health outcomes, but quantitative evidence across age groups and outcome types remains limited. This meta-analysis synthesizes data from 45 randomized controlled trials to assess the impact of painting-based art interventions on mental health. The study explores potential moderating factors such as intervention duration, type of art, gender, and age group. A rigorous quality assessment of included studies was performed using Cochrane’s risk of bias tool. The pooled effect size for painting-based interventions on mental health was significant, indicating a large positive impact. Subgroup analyses revealed that interventions of various durations, art forms, and gender compositions produced similar effects. Notably, older adults benefited the most from these interventions. Painting-based art interventions were associated with improved scale-assessed mental health outcomes. These findings should be interpreted as evidence for one visual art-making approach within broader art therapy practice, rather than as defining art therapy solely by painting-based methods.

## 1. Introduction

Mental health has been recognized as a serious public health problem that adds significantly to the world’s disease burden and affects the well-being of individuals, cohesion of communities and socio-economic development (Okesanya et al., 2025). Depression and anxiety, which are prevalent common mental health disorders, are on the rise, thus placing a burden on world health systems (Cuijpers et al., 2020). The psychological resilience of vulnerable groups like youth faces greater challenges during special periods such as the COVID-19 pandemic, and mental health problems become more prominent (Varma et al., 2021). Although drug therapy and conventional psychotherapy are the most commonly employed clinical interventions, their efficacy is still limited by a variety of factors, including adverse drug reactions, treatment costs, limited access to professional services, and patients’ unwillingness to seek help due to social stigma (Leichsenring et al., 2022). Therefore, it is important to explore and validate accessible, cost-effective, and low side-effect non-pharmacological interventions for mental health, including art intervention as one of the potential approaches.

In response to this, multiple nonpharmacological intervention strategies are being employed to improve life as well as give innovative psychological support. All this is backed by growing evidence-based medicine. One example is physical activity, “green exercise” in particular, which is shown to be an effective mental health and mood booster (Barton & Pretty, 2010; Sowa & Meulenbroek, 2012). In addition, mindfulness-based training improves the psychological qualities of an individual and thus is associated with better mental health outcomes (Quaglia et al., 2016). In light of increasing digital technologies in the last few decades, the introduction of AI-based conversational agents (H. Li et al., 2023) and various e-mental health interventions (Phillips et al., 2019) is being regarded as a potential remedy for enhancing service accessibility. Through the exploration of myriad strategies and tools, important lessons can be learnt in order to deal with global mental health problems.

Art intervention is starting to receive more attention amongst numerous non-pharmacological interventions. For example, painting-based activities represent one form of art therapy that can be used to facilitate emotional expression and patient engagement (Perkins, 2020). As individuals create works of art, they may transform internal experiences that are difficult to verbalize, such as emotional distress, tension, or fragmented feelings, into visible external symbols, and this process has the potential to integrate the psyche and regulate emotions (An, 2025). In recent years, many interventions in art have been aimed at very different kinds of people in diverse situations. For instance, as a result of the COVID-19 pandemic, one study used online art therapy to improve primary school mental health (Malboeuf-Hurtubise et al., 2021); in addition, recently, mindfulness-based art therapy was used to improve the mental state of early childhood educators (Ram-Vlasov, 2024). As technological advancements unfold, researchers have also started to study the use of artificial intelligence in order to aid the assessment process in art therapy (J. Kim et al., 2024), which forecasts the future innovation trend of this field.

Many empirical studies show the possibilities of getting good mental health by using art interventions, but the existing evidence is mostly heterogeneous. There are crucial differences between studies in terms of intervention protocols (e.g., type of art, duration of intervention), participant characteristics (e.g., age, baseline mental state), and outcome measurement tools, hampering the comparison and integration of findings across studies. In addition, while there is some research on painting interventions for specific populations (An, 2025; Kehr & Haeyen, 2024), there is still a dearth of systematic quantitative assessment of whether the effects of painting interventions differ by age group and gender group. Most of the earlier review works are qualitative summaries or address broader issues related to art interventions, rather than meta-analyses focusing on high-quality randomized controlled trials (RCTs) evaluating the effects of painting-based interventions as a subset of art therapy. Due to the current state of fragmented evidence, our understanding of the magnitude and robustness of painting interventions remains limited. This, in turn, hinders the translation of these findings into clinical practice and public health applications.

Thus, this research is specifically intended to fill this research gap through a meta-analysis of existing randomized controlled trials. The primary goals of this study are (1) to quantitatively assess the overall effect size of painting-based art interventions on validated scale-assessed mental health outcomes, including depression, anxiety, and psychiatric symptoms; (2) to systematically explore whether a series of potential moderating variables (i.e., intervention time, painting-based intervention form, gender ratio of participants, and age group) affect the intervention effect; and (3) to thoroughly evaluate the methodological quality of the studies that were included and to investigate potential publication bias. This study aims to provide a clear estimate of the effectiveness of art intervention as well as clarify under which conditions it is effective, by comprehensively and systematically synthesizing existing RCT evidence. The hope is to provide robust evidence-based support to clinicians, policymakers and future researchers. Further, to promote scientific developments and standardized applications of art intervention in mental health practices.

## 2. Materials and Methods

This meta-analysis included both international and Chinese-language literature, with CNKI (China National Knowledge Infrastructure) used as a major Chinese database. Therefore, the evidence base should be understood in a cross-cultural context, with a substantial representation of studies conducted in China. Because art therapy has strong roots in Western professional and theoretical traditions, painting-based art interventions in Chinese studies may not always correspond directly to Western art therapy practice as a regulated profession. In this research, “mental health outcomes” were operationally defined as scale-assessed psychological outcomes, including depression, anxiety, and psychiatric symptoms, measured using validated psychometric instruments.

### 2.1. Research Design and Registration

We systematically synthesize evidence from randomized controlled trials regarding the mental health effects of painting intervention, and conduct a systematic review and meta-analysis in strict accordance with PRISMA 2020 reporting guidelines (Page et al., 2021). The research protocol was pre-registered on the PROSPERO international prospective systematic review registration platform (registration number: CRD420261281858) prior to the official search to guarantee the transparency of the research.

### 2.2. Literature Search Strategies

This search was independently conducted by two researchers. The two researchers systematically searched PubMed, Web of Science, Scopus, Google Scholar, and CNKI from 2000 to September 2025. The strategy for searching in English is formulated by merging subject-specific terms with general free terms. Detailed information about the primary search terms can be found in Table S1. The search results were evaluated and screened by two independent researchers. The initial screening and secondary screening were conducted based on pre-set inclusion and exclusion criteria. If there were any disagreements, a consensus was reached through discussion.

### 2.3. Literature Screening and Inclusion/Exclusion Criteria

**Figure 1:**
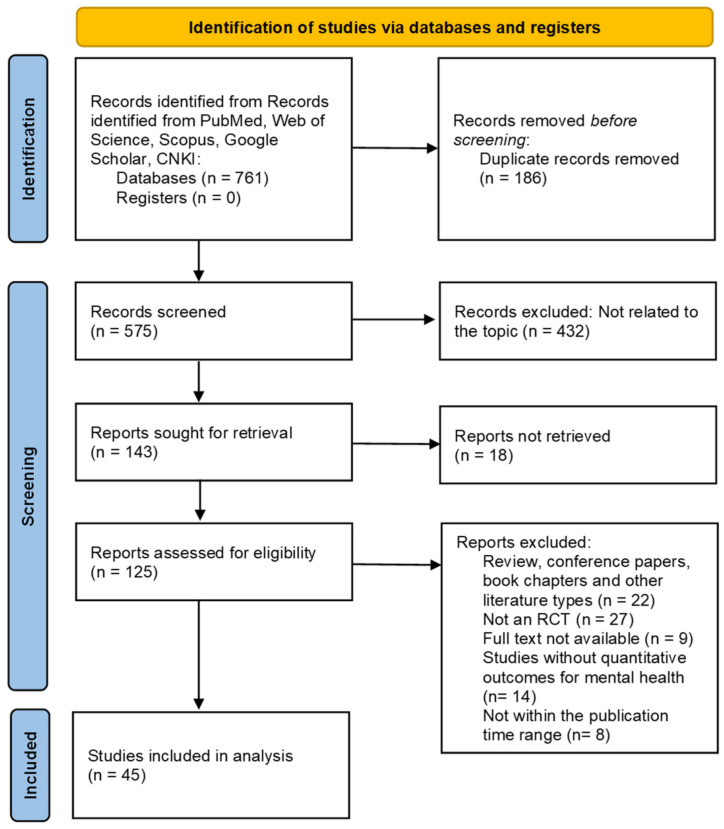
PRISMA flow diagram.

The PICOS framework served as the basis for the development of the literature screening criteria (Higgins et al., 2019). Inclusion criteria: The study participants are not limited by age, gender, or country/region, but are individuals with mental health problems (mental health problems are defined as symptoms such as depression, anxiety, stress, and psychological distress, as assessed by validated psychometric tools). The intervention measures were art interventions centered on painting, including non-directive free painting, mandala painting, theme-guided painting, and comprehensive art intervention. No restriction was placed on the specific painting medium; eligible studies could use watercolor, acrylic paint, markers, colored pencils, or other art materials, depending on the intervention design of the original study. Free painting refers to a non-directive form of painting in which participants were not given a fixed theme but were encouraged to paint images, feelings, or ideas that came to mind. The control group either underwent standard care or received no intervention. The outcome measures encompass mental health-related results evaluated using standardized scales, including assessments for depression and anxiety. The study design is a randomized controlled trial. The publication time is between 2000 and 2025. Exclusion criteria include: non-randomized controlled trials, case reports, review articles, conference abstracts, articles for which full text is unavailable, articles whose publication dates do not meet the requirements, and articles that have been retracted or have incomplete data. This study systematically presents the literature retrieval and screening steps using the PRISMA standardized process, detailing the changes in the number of documents at each stage and the corresponding exclusion criteria (Figure 1).

### 2.4. Data Extraction

The literature screening was done in a dual-person mode. In the first stage of screening, two researchers read the title and abstract separately and made a preliminary assessment based on the inclusion criteria; in the second stage of screening, the full text of the literature retained in the first stage was assessed. According to Borenstein, any disputes should be resolved by discussion, with third-party arbitration introduced if necessary. Through pilot trials on a small scale, data extraction was done using pre-designed and optimized standardized forms. The gathered information included fundamental characteristics of the research, such as the author, year of publication, country or region, and size of the sample, participant features: age; sex ratio; mental state in baseline, intervention characteristics: painting-based intervention form (no specific paint medium was restricted in the analysis); frequency of intervention; duration of each intervention; total period of intervention, control group features, outcome measurement tool, and statistical data (mean, standard deviation, sample size or other convertible data) for effect size.

### 2.5. Quality Assessment

The quality assessment of randomized controlled trials included in the studies was carried out using the Cochrane risk bias assessment tool (Sterne et al., 2019). The evaluation criteria include the generation of random sequences, ensuring allocation concealment, blinding both participants and researchers, obscuring outcome assessments, completeness of the outcome data, and selective reporting of outcomes, among other factors. Each dimension was classified as “low risk”, “uncertain”, or “high risk”. Two assessors independently completed a quality assessment, and disagreements were resolved by discussion or a third assessor. For data processing and generation of visualization charts, Review Manager (RevMan) 5.4 was used.

### 2.6. Data Synthesis and Analysis

The standardized mean difference (SMD) and its 95% confidence interval were utilized to facilitate comparisons across studies (Cohen, 2013). Priority was given to the extraction and analysis of post-intervention measurements. If a study reports only changes, you must convert the data using existing methods (Borenstein, 2024). The heterogeneity of population characteristics, intervention protocols, and outcome measurements between included studies warranted the use of a random-effects model to pool the effect sizes. Cochran’s Q test and the I^2^ statistic were used to evaluate the study’s heterogeneity (Higgins et al., 2003; Higgins & Thompson, 2002). Prediction intervals were calculated for all analyses to estimate the range of effects in similar studies in the future. To examine the sources of heterogeneity, pre-designed subgroup analyses were conducted according to intervention time, type of painting, gender ratio, and age. The elimination test (Viechtbauer & Cheung, 2010) was employed to examine the robustness of our findings, as well as a replication analysis of the large-sample and high-quality study. Comprehensive assessment of publication bias was performed by enhanced funnel plots, Egger regression tests, and trimming and complementation (Egger et al., 1997). All statistical analyses were accomplished in the R software (version 4.5.1) environment, and forest plots and funnel plots were also produced by R. Each statistical test was evaluated in both possible directions (two-tailed), and the significance level was set to α = 0.05, whilst the Q-test for the heterogeneity was set to α = 0.10.

## 3. Results

### 3.1. Identification of the Studies

Following our literature search strategy, we systematically searched five databases, including PubMed, Web of Science, Scopus, Google Scholar, and CNKI. A total of 761 records in the database were found. In order for the documented literature to stand on its own, 186 duplicate records were removed.

Figure 1 shows the literature selection process PRISMA flowchart. An analysis of 575 records was undertaken, and 432 documents that were irrelevant to the study were explicitly excluded. We excluded 18 for which a search record was not available; 125 were included for full-text evaluation. After a full-text evaluation of the 125 documents, 80 were excluded by the two reviewers. The detailed reasons for the exclusion are as follows: 22 were non-original research; 27 were non-RCTs; 9 could not be obtained in full text; 14 were missing quantitative data around mental health; and 8 were not published within the specified time frame. A total of 45 randomized controlled trials were included in the final analysis (Abbing et al., 2019; Akay et al., 2025; Bell & Robbins, 2007; Dai, 2015; Feng et al., 2020; Forouzandeh et al., 2020; Fu et al., 2021; Goodarzi et al., 2020; Gu et al., 2025; Guo et al., 2007; Gürcan & Atay Turan, 2021; Gussak, 2006, 2009; Hong et al., 2025; Y. Huang, 2020; Ilali et al., 2018; S. K. Kim, 2013; Köksal et al., 2025; D. Li et al., 2020; P. Li et al., 2017; Y. Li et al., 2022; Y. Li et al., 2017; M. Liu et al., 2024; Q. Liu et al., 2021; X. Liu et al., 2023; X. Liu & Lu, 2018; Lone et al., 2021; Lv et al., 2022; S. Ma, 2021; X. Ma et al., 2025; Masika et al., 2021; Meng et al., 2005; Montag et al., 2014; Niu & Cao, 2011; Rajendran et al., 2020; Ran et al., 2021; Sandmire et al., 2012; Shen, 2024; J. Sun et al., 2022; L. Sun et al., 2021; J. Xu & Wang, 2022; Y. Xu et al., 2022; Yuan, 2023; Zhao & Tang, 2017; Zhou & Zhang, 2022).

### 3.2. Literature Quality Assessment

**Figure 2:**
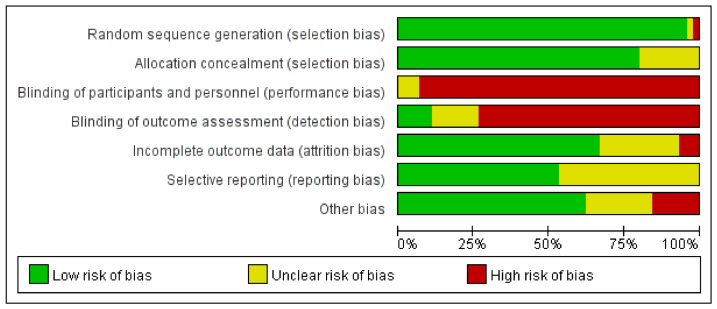
Risk of bias diagram.

The quality of the randomized controlled trials included in this study was assessed using the Cochrane risk of bias evaluation tool, and the results are shown in Figure 2. With respect to random sequence generation, most studies performed satisfactorily, while very few studies were judged high risk or did not satisfactorily report. In terms of allocation concealment, we rated around 75% of the studies assessed to be of low risk of bias. The participant and implementer blinding dimension showed significant limitations. Given the unique nature of the painting intervention, implementing a double-blind design faces inherent difficulties in practice; therefore, more than half of the studies were rated as high risk in this area. In contrast, most studies employed measures such as independent assessors or standardized scales to reduce detection bias in outcome assessment blinding. Data completeness was generally satisfactory, with only a few studies rated as high risk due to high dropout rates or missing intention-to-be-analyzed data. The assessment results for selective reporting bias and other biases showed that most studies were able to fully report the pre-specified outcome indicators, but some studies still had issues, such as inconsistencies between outcome indicators and the protocol or potential conflicts of interest.

### 3.3. Meta-Analysis of Overall Effects

**Figure 3:**
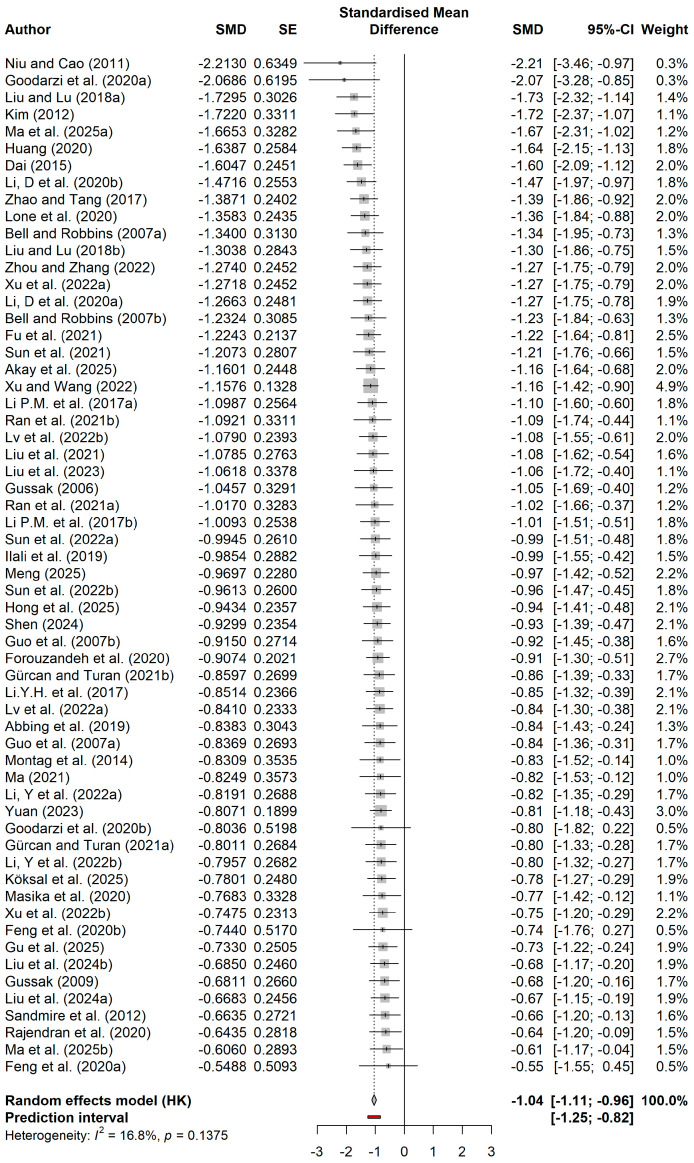
Forest plot of the effects of painting-based intervention on mental health. Data from: (Abbing et al., 2019; Akay et al., 2025; Bell & Robbins, 2007; Dai, 2015; Feng et al., 2020; Forouzandeh et al., 2020; Fu et al., 2021; Goodarzi et al., 2020; Gu et al., 2025; Guo et al., 2007; Gürcan & Atay Turan, 2021; Gussak, 2006, 2009; Hong et al., 2025; Y. Huang, 2020; Ilali et al., 2018; S. K. Kim, 2013; Köksal et al., 2025; D. Li et al., 2020; P. Li et al., 2017; Y. Li et al., 2017; Y. Li et al., 2022; M. Liu et al., 2024; Q. Liu et al., 2021; X. Liu et al., 2023; X. Liu & Lu, 2018; Lone et al., 2021; Lv et al., 2022; S. Ma, 2021; X. Ma et al., 2025; Masika et al., 2021; Meng et al., 2005; Montag et al., 2014; Niu & Cao, 2011; Rajendran et al., 2020; Ran et al., 2021; Sandmire et al., 2012; Shen, 2024; J. Sun et al., 2022; L. Sun et al., 2021; J. Xu & Wang, 2022; Y. Xu et al., 2022; Yuan, 2023; Zhao & Tang, 2017; Zhou & Zhang, 2022).

This study included 45 randomized controlled trials. The forest plot (Figure 3) clearly shows the effect size distribution and precision of each independent study. All included studies had negative standardized mean differences, with effect sizes ranging from −2.21 to −0.55, suggesting a consistent positive effect of art intervention on improving mental health outcomes. The analysis of pooled effect sizes indicated that the mental health measures in the group participating in the art intervention were considerably more favorable compared to the control group (SMD = −1.04, 95% CI = −1.11 to −0.96), achieving a substantial effect size as per Cohen’s criteria for effect sizes. The prediction interval was −1.25 to −0.82, indicating that even considering potential inter-study variability, future similar studies can still be expected to achieve clinically significant moderate to large effects, further confirming the statistical robustness of this effect. The I^2^ statistic was only 16.8% (*p* = 0.1375), suggesting that the variability among studies mainly stemmed from sampling error rather than actual effect differences, indicating high homogeneity among the included studies.

### 3.4. Subgroup Analysis Results

To further examine factors that may moderate the effects of the included interventions, subgroup analyses were conducted across four dimensions: mean age group, gender composition, painting-based intervention form, and intervention time. The “painting-based intervention form” subgroup was used to classify the primary art-making task reported in the included studies, such as mandala coloring/painting and theme-based or guided painting, rather than to define distinct schools or formal modalities of art therapy. A total of 45 studies were included in the meta-analysis. The results are summarized in Table 1.

**Figure 4:**
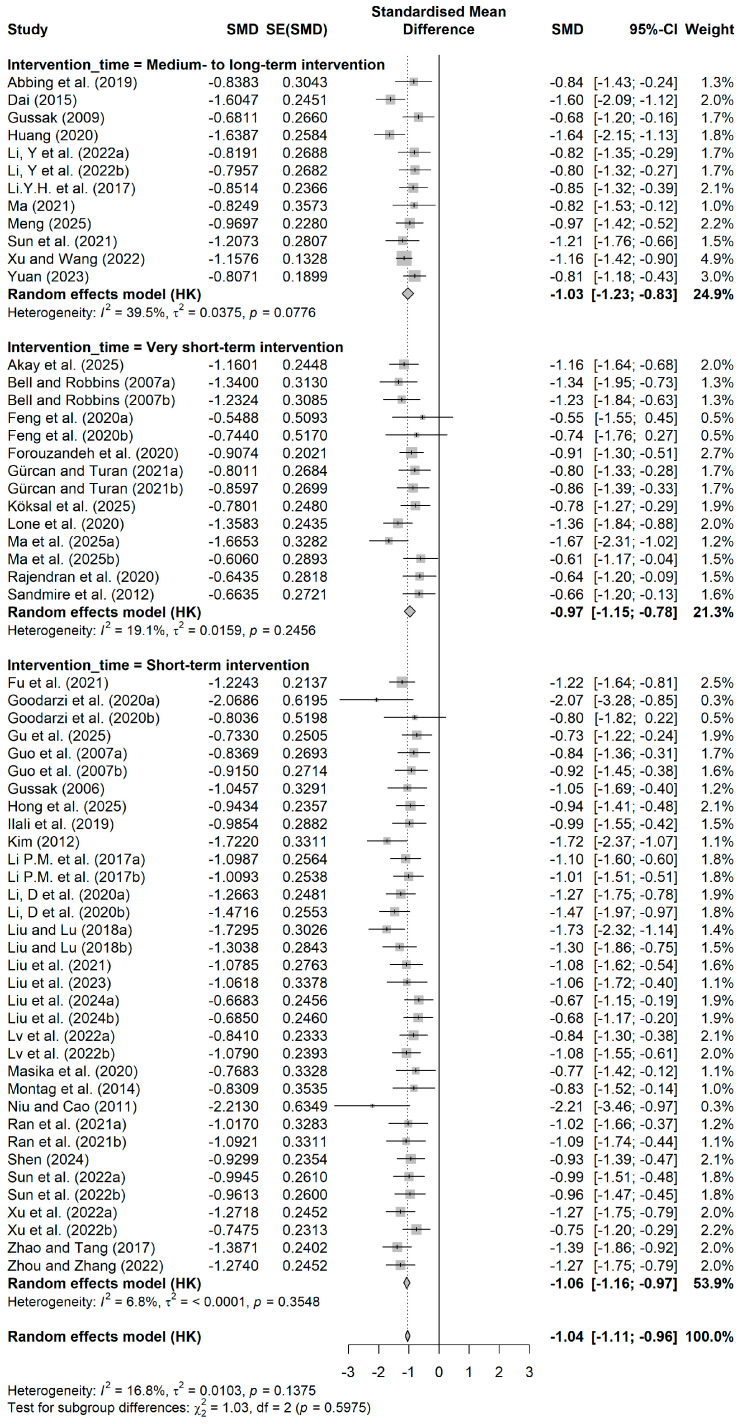
Forest plot by intervention time. Data from: (Abbing et al., 2019; Akay et al., 2025; Bell & Robbins, 2007; Dai, 2015; Feng et al., 2020; Forouzandeh et al., 2020; Fu et al., 2021; Goodarzi et al., 2020; Gu et al., 2025; Guo et al., 2007; Gürcan & Atay Turan, 2021; Gussak, 2006, 2009; Hong et al., 2025; Y. Huang, 2020; Ilali et al., 2018; S. K. Kim, 2013; Köksal et al., 2025; D. Li et al., 2020; P. Li et al., 2017; Y. Li et al., 2017; Y. Li et al., 2022; M. Liu et al., 2024; Q. Liu et al., 2021; X. Liu et al., 2023; X. Liu & Lu, 2018; Lone et al., 2021; Lv et al., 2022; S. Ma, 2021; X. Ma et al., 2025; Masika et al., 2021; Meng et al., 2005; Montag et al., 2014; Niu & Cao, 2011; Rajendran et al., 2020; Ran et al., 2021; Sandmire et al., 2012; Shen, 2024; J. Sun et al., 2022; L. Sun et al., 2021; J. Xu & Wang, 2022; Y. Xu et al., 2022; Yuan, 2023; Zhao & Tang, 2017; Zhou & Zhang, 2022).

Figure 4 shows the forest plot by intervention time. Regarding intervention time, short-term interventions were the most numerous (k = 34, SMD = −1.06), with a pooled effect size of −1.06 (95% CI = −1.16~−0.97), indicating low heterogeneity (I^2^ = 6.80%). Mid- to long-term interventions (k = 12, SMD = −1.03) and very short-term interventions (k = 14, SMD = −0.97) also showed significant improvement, with all three groups reaching the large effect criterion. No statistically significant differences were found between groups (Q = 1.03, *p* = 0.5975), suggesting that the duration of the intervention did not substantially affect the mental health benefits of the painting intervention.

**Figure 5:**
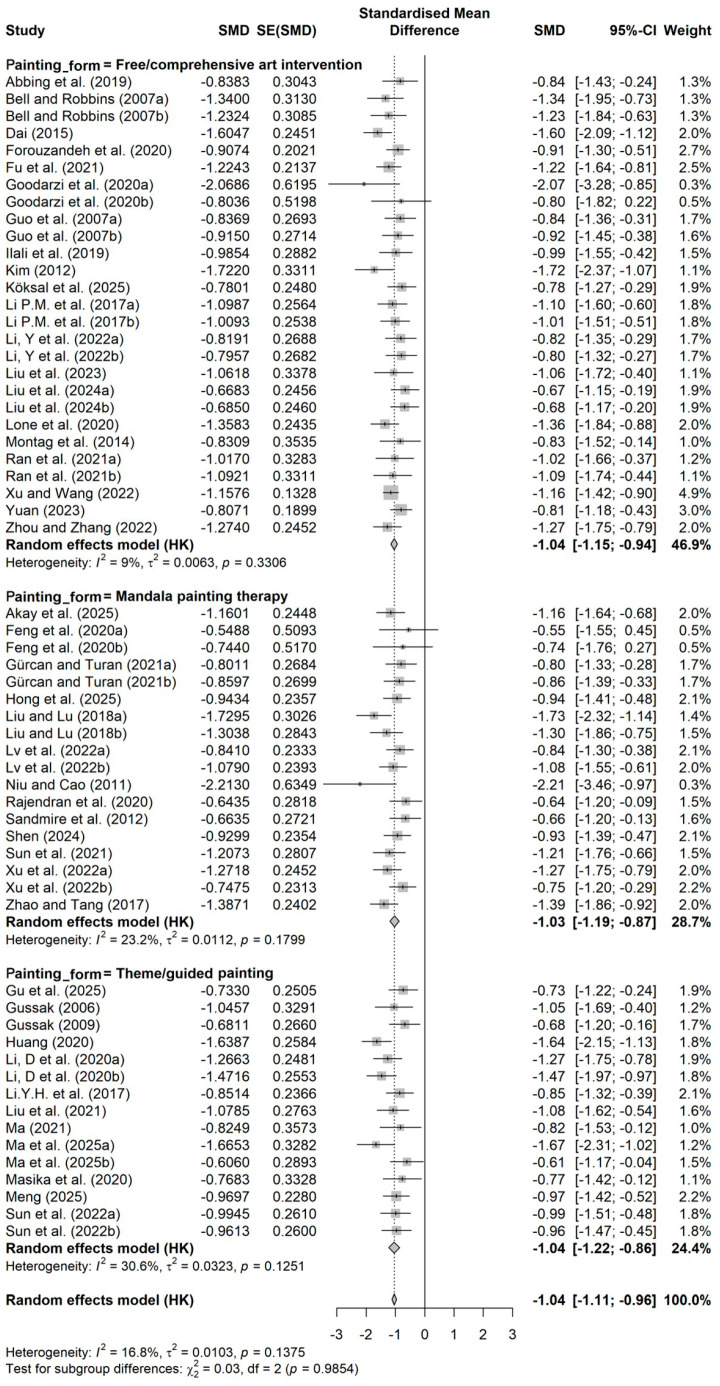
Forest plot by painting-based intervention form. Data from: (Abbing et al., 2019; Akay et al., 2025; Bell & Robbins, 2007; Dai, 2015; Feng et al., 2020; Forouzandeh et al., 2020; Fu et al., 2021; Goodarzi et al., 2020; Gu et al., 2025; Guo et al., 2007; Gürcan & Atay Turan, 2021; Gussak, 2006, 2009; Hong et al., 2025; Y. Huang, 2020; Ilali et al., 2018; S. K. Kim, 2013; Köksal et al., 2025; D. Li et al., 2020; P. Li et al., 2017; Y. Li et al., 2017; Y. Li et al., 2022; M. Liu et al., 2024; Q. Liu et al., 2021; X. Liu et al., 2023; X. Liu & Lu, 2018; Lone et al., 2021; Lv et al., 2022; S. Ma, 2021; X. Ma et al., 2025; Masika et al., 2021; Meng et al., 2005; Montag et al., 2014; Niu & Cao, 2011; Rajendran et al., 2020; Ran et al., 2021; Sandmire et al., 2012; Shen, 2024; J. Sun et al., 2022; L. Sun et al., 2021; J. Xu & Wang, 2022; Y. Xu et al., 2022; Yuan, 2023; Zhao & Tang, 2017; Zhou & Zhang, 2022).

The moderating effect analysis of the painting-based intervention form revealed a similar pattern. Figure 5 is the forest plot by painting-based intervention form. Free/comprehensive art intervention had the most extensive inclusion in studies (k = 27, SMD = −1.04), with low intra-group heterogeneity (I^2^ = 9.00%). Mandala painting therapy (k = 18, SMD = −1.03) and theme/guided painting (k = 15, SMD = −1.04) had comparable effect sizes, and the 95% confidence intervals of the three intervention forms largely overlapped. Inter-group comparisons showed almost no differences between subgroups (Q = 0.03, *p* = 0.9854), indicating that the intervention effect remained stable regardless of the painting form used.

**Figure 6:**
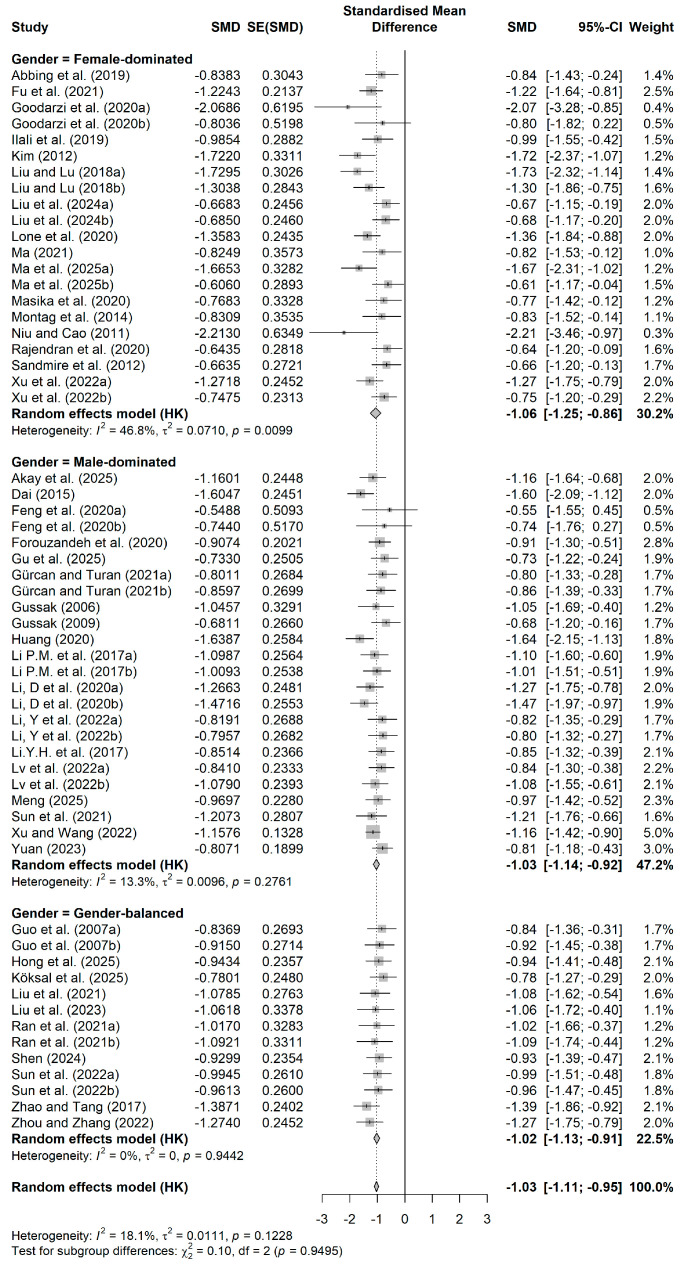
Forest plot by gender. Data from: (Abbing et al., 2019; Akay et al., 2025; Dai, 2015; Feng et al., 2020; Forouzandeh et al., 2020; Fu et al., 2021; Goodarzi et al., 2020; Gu et al., 2025; Guo et al., 2007; Gürcan & Atay Turan, 2021; Gussak, 2006, 2009; Hong et al., 2025; Y. Huang, 2020; Ilali et al., 2018; S. K. Kim, 2013; Köksal et al., 2025; D. Li et al., 2020; P. Li et al., 2017; Y. Li et al., 2017; Y. Li et al., 2022; M. Liu et al., 2024; Q. Liu et al., 2021; X. Liu et al., 2023; X. Liu & Lu, 2018; Lone et al., 2021; Lv et al., 2022; S. Ma, 2021; X. Ma et al., 2025; Masika et al., 2021; Meng et al., 2005; Montag et al., 2014; Niu & Cao, 2011; Rajendran et al., 2020; Ran et al., 2021; Sandmire et al., 2012; Shen, 2024; J. Sun et al., 2022; L. Sun et al., 2021; J. Xu & Wang, 2022; Y. Xu et al., 2022; Yuan, 2023; Zhao & Tang, 2017; Zhou & Zhang, 2022).

In the subgroup analysis by gender ratio (Figure 6), the painting-based art intervention showed comparable beneficial effects across female-dominated, male-dominated, and gender-balanced samples. The female-dominated subgroup showed a slightly larger pooled effect than the other two subgroups, although the difference was minimal and not statistically significant. Specifically, the pooled effects were similar in the female-dominated group (k = 21, SMD = −1.06), the male-dominated group (k = 24, SMD = −1.03), and the gender-balanced group (k = 13, SMD = −1.02). Heterogeneity was absent in the gender-balanced subgroup (I^2^ = 0.00%), suggesting a high degree of consistency across studies in this subgroup. In contrast, the female-dominated subgroup showed moderate intra-group heterogeneity (I^2^ = 46.80%). The between-subgroup test was not statistically significant (Q = 0.10, *p* = 0.9495), indicating that sample sex composition had little influence on the intervention effect. One study was excluded from this subgroup analysis because it did not report the gender ratio.

**Figure 7:**
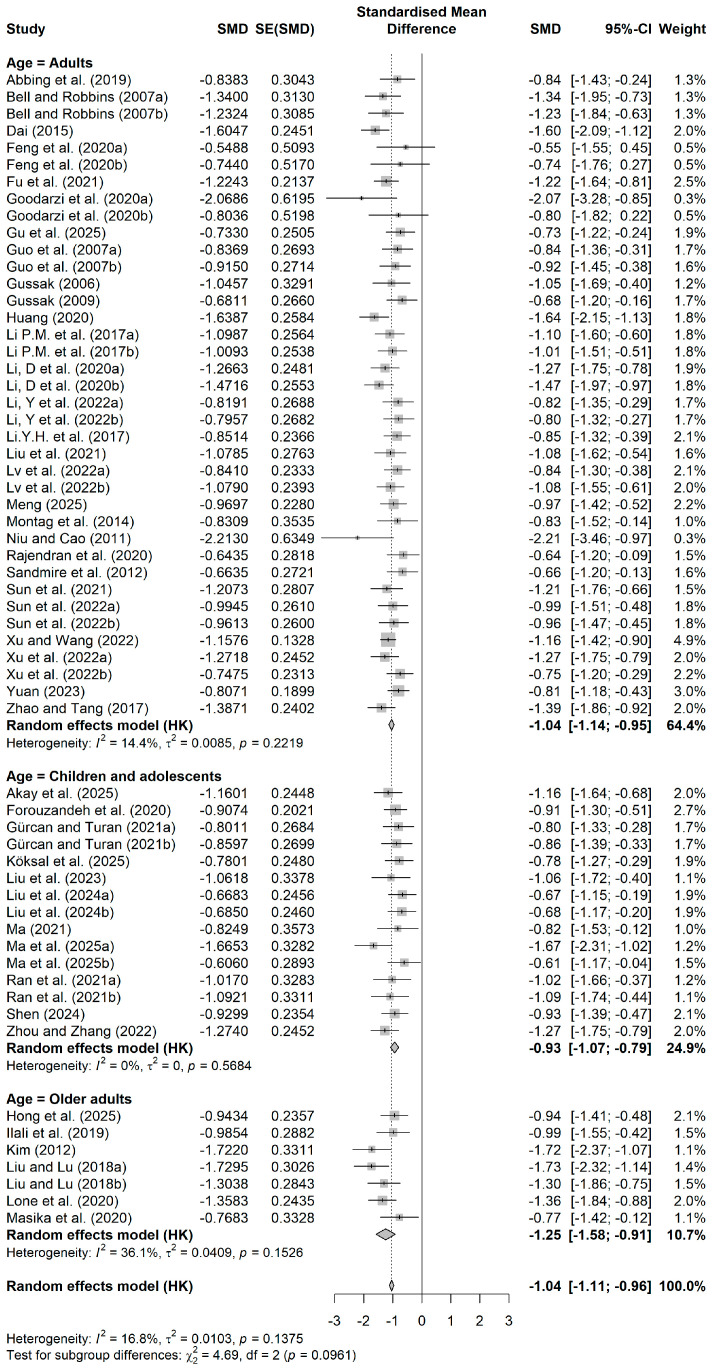
Forest plot by average age. Data from: (Abbing et al., 2019; Akay et al., 2025; Bell & Robbins, 2007; Dai, 2015; Feng et al., 2020; Forouzandeh et al., 2020; Fu et al., 2021; Goodarzi et al., 2020; Gu et al., 2025; Guo et al., 2007; Gürcan & Atay Turan, 2021; Gussak, 2006, 2009; Hong et al., 2025; Y. Huang, 2020; Ilali et al., 2018; S. K. Kim, 2013; Köksal et al., 2025; D. Li et al., 2020; P. Li et al., 2017; Y. Li et al., 2017; Y. Li et al., 2022; M. Liu et al., 2024; Q. Liu et al., 2021; X. Liu et al., 2023; X. Liu & Lu, 2018; Lone et al., 2021; Lv et al., 2022; S. Ma, 2021; X. Ma et al., 2025; Masika et al., 2021; Meng et al., 2005; Montag et al., 2014; Niu & Cao, 2011; Rajendran et al., 2020; Ran et al., 2021; Sandmire et al., 2012; Shen, 2024; J. Sun et al., 2022; L. Sun et al., 2021; J. Xu & Wang, 2022; Y. Xu et al., 2022; Yuan, 2023; Zhao & Tang, 2017; Zhou & Zhang, 2022).

The average age-based forest plot is shown in Figure 7. The significant impact magnitude was seen in the elder population (k = 7, SMD = −1.25, 95% CI = −1.58 to −0.91), followed by the adult population (k = 38, SMD = −1.04), although the number of studies was low. The effect size of the children and adolescents’ population (k = 15, SMD = −0.93) was relatively small but still clinically significant. The test for intergroup difference shows the *p* = 0.0961, not significant in the traditional sense, but still showing a borderline significance, which shows that perhaps age influences the effect of the painting intervention, as older people seem to benefit more significantly. This result needs to be verified in the future.

### 3.5. Sensitivity Analysis

In order to check the robustness of the findings of this meta-analysis, a sensitivity analysis was performed on outlier removal (Table 2). The initial 145 effect sizes produced a pooled effect of SMD = −1.32 (95% CI = −1.52 to −1.12, *p* < 0.0001), but they included a very high degree of heterogeneity (I^2^ = 93.00%, Q = 2049.95).

Through a systematic elimination strategy involving 85 outlier effect sizes, the pooled result of the remaining 60 effect sizes showed SMD = −1.04 (95% CI = 1.11 to −0.96, *p* < 0.0001). After eliminating outliers, the heterogeneity reduced significantly, whereby I^2^ dropped from 93.00% to 16.80% with the Q statistic decreasing from 2049.95 to 70.92, indicating remaining studies’ heterogeneity was due largely to sampling error. Significant contributions of outliers to heterogeneity expansion in the first analysis are confirmed by these results.

The outcome of a trim-and-fill analysis also confirmed the above findings. There is no need to add any missing studies (the adjusted addition value shows 0), and the adjusted effect size is the same as after an outlier removal (SMD = −1.04, 95% CI = −1.11 to −0.96). Both sensitivity analysis approaches pointed to a stable effect estimate. In short, the effect size decreased after removing the outlier; however, it still falls in the large effect size category, and its statistical significance did not alter. This is a strong demonstration that the painting intervention has a positive effect on mental health, and not some outliers or extreme studies. This is a robust phenomenon widespread in studies.

### 3.6. Publication Bias Test

**Figure 8:**
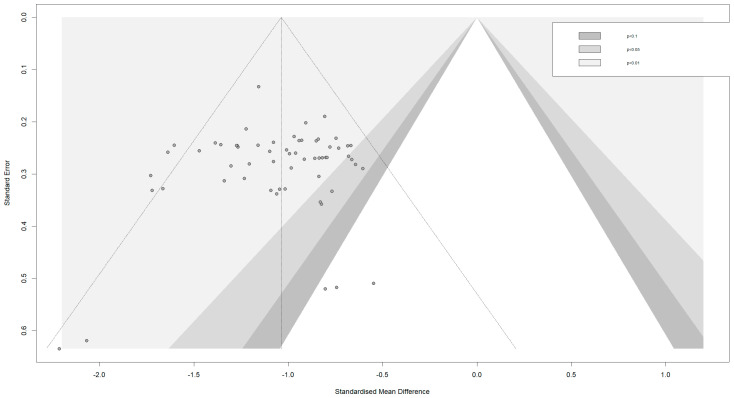
Publication bias funnel plot (Each dot represents an individual study. The vertical line indicates the pooled effect size (SMD = −1.04), and the diagonal lines represent the pseudo 95% confidence limits).

An enhanced funnel plot was utilized in this research to visually evaluate possible publication bias. Figure 8 illustrates that the funnel plot, featuring the standardized mean difference on the horizontal axis and the standard error on the vertical axis, exhibits a fairly consistent inverted funnel shape. The included studies are distributed around both sides of the pooled effect size (SMD = −1.04), and the overall symmetry is acceptable.

Looking at the specific characteristics of the study distribution, the vast majority of effect sizes fall within the statistical significance region (*p* < 0.05 and *p* < 0.01), and are concentrated in the range of −0.5 to −1.5. Studies with higher precision (smaller standard errors) cluster closely around the pooled effect size at the top of the funnel, while studies with lower precision (larger standard errors) exhibit the expected more dispersed distribution pattern—a pattern consistent with theoretical expectations of random sampling error. There are limited studies on the right-hand side of the funnel plot. The fact that unsuccessful studies are not published is a possibility in this context. Nevertheless, because art-based intervention studies often report beneficial outcomes, the observed distribution may represent the true range of intervention effects rather than being solely attributable to publication bias.

## 4. Discussion

This research examined 45 randomized controlled trials to look at the influence of painting-based art intervention on mental health. The findings indicated that painting-based art intervention was beneficial in improving the mental health indicators. In particular, SMD = −1.04 (95% CI = 1.11 to −0.96) was the pooled effect size. It suggests that painting-based art intervention may be able to promote mental health in clinical settings. The consistency of the studies included in this study was also beneficial due to the low level of heterogeneity (I^2^ = 16.8%).

Subgroup analyses revealed several significant trends. Regarding the dimension of intervention time, all the interventions, whether of short-term (SMD = −1.06), medium- and long-term (SMD = −1.03) or very short-term (SMD = −0.97), showed a significantly large effect size and there were no differences that were statistically significant. These findings suggest that painting-based art interventions may be beneficial across different intervention durations. In this study, intervention duration was categorized as very short-term intervention (single-session or ≤1 week), short-term intervention (2–8 weeks), and medium- to long-term intervention (≥9 weeks). All three duration subgroups showed significant effects, and no statistically significant differences were observed between them. Therefore, even relatively brief painting-based intervention programs may be beneficial for mental health, although further research is still needed to determine the optimal total intervention period and session length. Secondly, regarding the type of artwork utilized, the effect sizes of free creation/integrated art intervention, mandala painting therapy, and thematic/guided painting were very similar (all approximately SMD = −1.04). This indicates that different artworks are equivalent in promoting mental health, thus providing the basis for the flexible selection of intervention forms in clinical practice. What is more, the gender composition of the groups had little moderating effect on the intervention effect; the effect sizes of male-dominated, female-dominated, and gender-balanced groups were all at similar levels, suggesting the universality of art interventions. The age-group moderating effect analysis indicated a near-significant trend. The effect size for the older group was largest (SMD = −1.25). This was significantly higher than that for adults (SMD = −1.04) and children and adolescents (SMD = −0.93). Thus, an older individual may benefit more from the painting intervention.

Sensitivity analyses and assessments of publication bias provided additional support for the robustness of these results. The effect size remained stable after outlier removal, and the funnel plot showed a relatively symmetrical distribution pattern; these pieces of evidence collectively support the reliability of the study’s conclusions. The effect size found in this study (SMD = −1.04) is significantly higher than the results of several recently published meta-analyses of visual arts therapy (Joschko et al., 2024). The systematic review and meta-analysis included 69 studies, reporting pooled effect sizes of SMD = 0.38 (baseline change analysis) and SMD = 0.19 (post-test analysis), indicating a generally lower quality of research. The higher effect size in this study may be due to: first, the study focused on painting as a specific visual art form, rather than encompassing all types of visual arts therapy; second, the overall methodological quality of the included studies was moderate, with good randomization and data integrity; and third, the study population was predominantly from China, and the homogeneity of cultural background may have reduced the sources of heterogeneity.

Regarding research on art interventions in older adults, a meta-analysis by Quinn et al. (2025) examined the effects of group art interventions on depression and anxiety in older adults (>55 years). Research indicated that art interventions conducted in groups correlated with a significant decrease in depression levels (Cohen’s d = 0.70, 95% CI = 0.54–0.87), and the effect size for anxiety was also moderate (d = 0.76). In this study, the effect size of the older adult subgroup (SMD = −1.25) was larger than the reported value in the study. This difference may be attributable to the specific nature of the intervention, as this study focused exclusively on painting-based approaches, while Quinn et al.’s research covered a variety of art forms, including dance and music.

As for children and adolescents, a recent meta-analysis on the effects of art intervention on depressive symptoms found that the pooled effect size of 12 randomized controlled trials was SMD = −0.72 (95% CI = −1.28~−0.16) (B. Zhang et al., 2025). The effect size of the children and adolescents’ subgroup in this study (SMD = −0.93) was similar to but slightly higher than that of the other trials, and the heterogeneity was zero (I^2^ = 0%), showing higher research consistency.

Compared to traditional psychotherapy, art intervention showed encouraging effect sizes. Meta-analysis of cognitive behavioral therapy (CBT), a first-line psychotherapy for depression, showed an effect size of approximately Hedges’ g = 0.71 relative to the control group (adjusted for publication bias, g = 0.53) (Cuijpers et al., 2013). In this study, the effect size of the painting intervention (SMD = −1.04) was comparable to or even higher than that of CBT, indicating that painting therapy has the potential to be an effective complement to traditional psychotherapy. For anxiety, a meta-analysis by Bhattacharya et al. (2023) found that CBT had smaller effect sizes compared to placebo (Hedges’ g = 0.24). This goes on to testify to the efficacy of art intervention in helping with mental health.

Interventions utilizing specific artistic forms have been shown to be effective. A recent systematic review of mandala art intervention, a specific type of art therapy that involves creating art within a circular structure, concluded that mandalas may be effective in alleviating negative symptoms, increasing hope, relieving pain, and reducing specific physiological indicators of stress (M. Q. Zhang et al., 2024). The mandala painting therapy subgroup, which was included in this study, had a moderate effect size (SMD = −1.03). This provides evidence for the effectiveness of the result.

In a meta-analysis including 35 studies involving 4071 participants, W. Huang et al. (2025) reported that visual art therapy was effective in reducing anxiety (SMD = –1.31, 95% CI = –1.80 to –0.95). The size effect resembles the findings of this research, where SMD = −1.04. Huang et al.’s study mainly examined anxiety symptoms, not other mental health indicators. This means that the results of the two studies are mutually reinforcing, together supporting the visual art intervention as effective for improving mental health.

It should be noted that this review does not define art therapy as a painting-based intervention alone. Contemporary art therapy is a broader professional and theoretical field in which art therapists select materials, media, and intervention methods according to clients’ needs, treatment goals, clinical context, and therapeutic relationships. Therefore, the present findings should be interpreted as evidence regarding painting-based art interventions as one specific visual art-making approach, rather than as evidence for the whole field of art therapy.

### 4.1. Limitations

This meta-analysis carries a few shortcomings that must be noted. First, at the methodological level, this study’s main weakness is the limitation of blinding participants and implementers. Due to the nature of painting interventions, it is challenging to implement a double-blind design in practice. More than half of the studies were rated as high-risk on this front. Additionally, there was moderate implementation of allocation concealment, although nearly half of the studies reported poorly or had methodological shortcomings. To a certain degree, these methodological limitations can hinder accurate effect size estimation. Secondly, there is an imbalance in the geographical distribution of the involved studies, and most of the involved studies were completed in China, which is possibly limiting the universality of the research conclusions in other cultures. The different cultures background participants have different attitudes towards the expression of art; whether participants accept the activity of painting, whether they understand the meaning of mental health; all this needs to be verified to determine whether it will affect the effectiveness of the painting intervention.

The studies included in this meta-analysis assessed several types of mental health outcomes using validated psychometric scales, including depression, anxiety, and psychiatric symptoms. However, due to variability in outcome measures and the limited number of studies for some specific mental health conditions, we did not conduct subgroup analyses by type of mental health problem. Future studies should further examine whether the type of mental health condition moderates the effects of painting-based art interventions.

The overall level of heterogeneity was low in this research, but in some subgroups (i.e., the female-dominated group and the older adult group), there was considerable heterogeneity, which indicates potentially greater heterogeneity in these subgroups. Moreover, a large number of outlier effect sizes were removed in the first analysis, and both sets of results were robust in the sensitivity analysis, yet revealed considerable levels of heterogeneity in the original data. In this study, since the focus was mainly on the short-term benefit of the painting intervention, there was no adequate information to prove the long-term sustainability of these effects. Most included studies only assessed the effects immediately after the intervention ended, lacking follow-up data to examine the maintenance of the effects.

Furthermore, the tools used to measure mental health outcomes vary considerably across different studies. While standardized mean difference pooling analysis mitigated this issue to some extent, differences in mental health measurements across different scales can still increase the complexity of outcome interpretation. Finally, although publication bias was generally manageable, the relative scarcity of studies on the right side of the funnel plot (the region where effect sizes approach 0) suggests that under-publication of studies targeting negative outcomes cannot be completely ruled out.

### 4.2. Future Research Directions

Considering the results and constraints identified in this study, subsequent research could investigate the following areas more thoroughly. First, to confirm the universality and cultural sensitivity of painting interventions, it is essential to carry out additional high-quality, multi-center, and cross-cultural randomized controlled trials. Second, future research should strengthen the long-term tracking and evaluation of the effects of painting intervention. It is advisable to plan research protocols with follow-up periods of 3 months, 6 months, or even longer to systematically assess the persistence of intervention effects, and what might explain the maintenance of these effects.

Findings indicate that painting interventions may be particularly beneficial for older adults, a trend that merits further exploration. Through further studies, characteristics of older adults (cognitive function status, level of social support, and prior art experience) can be further refined to see which subgroups of older adults are more likely to benefit from painting intervention and the underlying mechanisms behind the enhanced intervention effects. Also, how painting-based interventions work to improve mental health is not clear. Future research may draw on frameworks from psychology, neuroscience, and art therapy to explore the roles of potential mediators, such as emotion regulation, self-expression, mindfulness experience, and social connection, in the development of a stronger theoretical model.

As digital technology develops, digital painting platforms and virtual reality-assisted art creation could produce painting intervention artworks. Future research may determine the feasibility and efficacy of these upcoming therapies. This is especially the case for those with limited mobility. Finally, due to the low cost and accessibility of art interventions, future research should consider incorporating health economics evaluations to assess their cost-effectiveness and inform policies and resource allocation.

## 5. Conclusions

This systematic review and meta-analysis of 45 randomized controlled trials assessed the overall effectiveness of painting-based art interventions on scale-assessed mental health outcomes. The available evidence suggests that these interventions may help improve mental health symptoms, particularly depression and anxiety. The effects were relatively stable across studies with different intervention durations, painting forms, and gender compositions. Subgroup analysis further suggested that older adults may experience relatively greater benefits, although this finding requires further confirmation through larger and multicenter studies. Overall, these findings support painting-based art interventions as a potentially useful non-pharmacological approach for improving mental health outcomes. However, they should be interpreted as evidence for one specific visual art-making approach within broader art therapy and arts-based intervention practice, rather than as representing art therapy.

Several uncertainties remain within the current evidence base. The findings were affected by methodological limitations, including insufficient blinding in some included studies, geographically concentrated samples, and short follow-up periods. Therefore, the current conclusions mainly reflect short-term intervention effects and are insufficient for determining long-term efficacy or stable mechanisms of action. In addition, differences in outcome measurement tools and intervention protocols across studies may complicate the interpretation of findings.

Although RCTs are useful for estimating intervention effects, they should be understood as only one form of evidence for painting-based art interventions. Future research should combine randomized controlled trials with qualitative, mixed-methods, and practice-based studies to better capture therapeutic processes, individualized clinical decision-making, and mechanisms of change. Further multicultural research is also needed to clarify the applicability, durability, and contextual adaptation of painting-based art interventions in diverse populations, and to examine how such interventions can be integrated into broader art therapy models according to client needs, clinical contexts, and therapeutic goals.

## Figures and Tables

**Table 1:** Subgroup analysis of painting-based intervention on mental health.

Variables	Subgroup	k	SMD	*p*-Value	95% CI	I^2^	tau^2^	Q
Intervention time	Medium- to long-term (≥9 weeks)	12	−1.0296	<0.0001	[−1.2290; −0.8302]	39.5%	0.0375	18.18
	Very short-term (Single-session/≤1 week)	14	−0.9656	<0.0001	[−1.1491; −0.7822]	19.1%	0.0159	16.07
	Short-term (2–8 weeks)	34	−1.0645	<0.0001	[−1.1637; −0.9652]	6.8%	<0.0001	35.42
Painting-based intervention form	Free/comprehensive art intervention	27	−1.0434	<0.0001	[−1.1515; −0.9353]	9%	0.0063	28.58
	Mandala painting therapy	18	−1.0277	<0.0001	[−1.1857; −0.8697]	23.2%	0.0112	22.13
	Theme/guided painting	15	−1.0388	<0.0001	[−1.2209; −0.8567]	30.6%	0.0323	20.16
Gender	Female-dominated	21	−1.0553	<0.0001	[−1.2501; −0.8606]	46.8%	0.071	37.62
	Male-dominated	24	−1.0298	<0.0001	[−1.1419; −0.9177]	13.3%	0.0096	26.54
	Mixed gender	13	−1.0213	<0.0001	[−1.1290; −0.9137]	0%	0	5.38
Average age	Adults	38	−1.0431	<0.0001	[−1.1376; −0.9486]	14.4%	0.0085	43.25
	Children and adolescents	15	−0.9348	<0.0001	[−1.0748; −0.7947]	0%	0	12.47
	Older adults	7	−1.2463	<0.0001	[−1.5805; −0.9121]	36.1%	0.0409	9.39

Note: One study did not report the gender, so it was excluded from the gender subgroup analysis.

**Table 2:** Sensitivity analysis results.

	Excluding/Adding Study	k	SMD	*p*-Value	95% CI	I^2^	H	Q
Observed values		145	−1.3238	<0.0001	[−1.5249; −1.1226]	93%	3.77	2049.95
Excluding outlier values	85	60	−1.0376	<0.0001	[−1.114; −0.9613]	16.8%	1.1	70.92
Adjustment (Adding) values	0	60	−1.0376	<0.0001	[−1.114; −0.9613]	16.8%	1.1	70.92

## Data Availability

The data supporting the findings of this study are available from the corresponding author upon reasonable request.

## Supplementary Materials

The following supporting information can be downloaded at: https://www.mdpi.com/article/10.3390/bs16050830/s1, Table S1: Strings used for the publications search.
