# Supplementary material for: Effects of Painting-Based Art Interventions on Mental Health Outcomes: A Meta-Analysis of Randomized Controlled Trials
Source: Behav Sci (Basel). 2026 May 21;16(5):830. doi: 10.3390/bs16050830 (PMC13203423; doi:10.3390/bs16050830)
Supplement: Supplementary file 1 [file behavsci-16-00830-s001.zip › behavsci-4283585-supplementary.pdf]

Supplementary Materials

Supplementary Table S1. Strings used for the publication search.

**Database**	**Search Strings**
PubMed	("Mental Health"[Mesh] OR "mental health" OR "psychological well-being" OR depression OR anxiety OR stress) AND ("Art Therapy"[Mesh] OR "drawing-based art therapy" OR "drawing therapy" OR "art therapy") AND ("Randomized Controlled Trial"[Publication Type] OR "RCT" OR "randomized controlled trial").
Wed of Science	TS = ("drawing-based art therapy" OR "drawing therapy" OR "art therapy") AND TS = ("mental health" OR "psychological well-being" OR depression OR anxiety OR stress) AND TS = ("randomized controlled trial" OR "RCT").
Scopus	(TITLE-ABS-KEY("drawing-based art therapy" OR "drawing therapy" OR "art therapy")) AND (TITLE-ABS-KEY("mental health" OR "psychological well-being" OR depression OR anxiety OR stress)) AND (TITLE-ABS-KEY("randomized controlled trial" OR "RCT")).
Google Scholar	"("drawing" OR "painting") therapy" AND "art therapy" AND "art intervention" AND "mental health" AND ("randomized controlled trial" OR RCT).
CNKI	摘要 = ("绘画" OR "绘画治疗" OR "绘画疗法") OR 摘要 = ("心理健康" OR "抑郁" OR "焦虑" OR "福祉") AND 摘要 = ("随机对照试验" OR "RCT").
